# Masse pré-rénale d'origine surrénalienne: quel est votre diagnostic?

**DOI:** 10.11604/pamj.2014.17.154.4089

**Published:** 2014-03-03

**Authors:** Hind El Aassri, Nawal El Ansari

**Affiliations:** 1 Service d'Endocrinologie, Diabétologie et Maladies métaboliques. Laboratoire de recherche PCIM, Faculté de Médecine et de Pharmacie, Université Quadi Ayyad, Marrakech, Maroc

**Keywords:** Masse pré-rénale, Phéochromocytome, IRM, pre-renal mass, pheochromocytoma, MRI

## Image en medicine

Le scanner ou l'imagerie par résonance magnétique (IRM) sont les examens de première intention afin de localiser une tumeur surrénalienne. L'imagerie doit d'abord explorer les glandes surrénales avec étude des deux jambages de la glande et la recherche de processus tumoral. En effet, pour préciser si la tumeur est d'origine surrénalienne ou pas, il suffit d’étudier ses rapports et son contact avec la glande; en présence d'une tumeur de l'aire surrénalienne refoulant la surrénale, elle est plutôt extrasurrénalienne, et si elle la déforme, la tumeur peut être considérée à priori comme étant d'origine surrénalienne. Nous rapportons le cas d'un patient de 27 ans, ayant comme antécédent un accident vasculaire cérébral ischémique remontant à un an faisant suite à un pic hypertensif. Le diagnostic d'hypertension artérielle a été depuis lors posé. Devant l’âge jeune du patient, un bilan d'hypertension artérielle secondaire a été réalisé, il avait objectivé une élévation significative des dérivés méthoxylés urinaires et du cortisol libre urinaire. Un scanner surrénalien a été, par la suite réalisé, il avait montré des surrénales d'aspect normal avec présence d'une lésion pré rénale gauche au contact du jambage externe de la surrénale mesurant 43 mm; elle est de forme ovalaire bien limitée spontanément hypodense (A), cette masse se réhausse d'une façon intense et hétérogène après injection de produit de contraste (B). Les coupes sagittales avaient bien montré la localisation pré-rénale de la masse dont le grand axe sagittal est de 54 mm (C) Le diagnostic d'un paragangliome du hile rénal a été évoqué en premier devant l'aspect radiologique et l’élévation des dérivés méthoxylés urinaires, mais il ne pourra pas expliquer l’élévation du cortisol libre urinaire. Le deuxième diagnostic évoqué était un phéochromocytome multisecrétant même si l'image radiologique n’était pas en faveur d'une tumeur d'origine surrénalienne mais le fait qu'elle arrivait au contact du jambage externe de la surrénale peut être un élément en faveur. Enfin, le diagnostic d'un corticosurrénalome multisecrétant ne peut être éliminé malgré l'absence de signes d'envahissement des structures de voisinage. Le patient a été adressé en chirurgie pour exérèse tumorale après une bonne préparation médicale à la chirurgie. Le diagnostic de phéochromocytome a été confirmé par l’étude anatomopathologique. L’évolution a été marquée par la régression de toute la symptomatologie. Les hypertensions artérielles (HTA) secondaires correspondent à moins de 10% des HTA tout-venant. Parmi celles-ci, les HTA d'origine surrénalienne qui correspondent à environ 3% des HTA avérées. Le phéochromocytome est une cause rare d'hypertension artérielle dont la prévalence chez l'hypertendu est estimée entre 0,1 et 0,6%, il peut être génétique ou sporadique. Le traitement chirurgicale s'impose devant tout phéochromocytome et le suivi doit être fait à long terme pouvant aller jusqu’à dix ans de suivi pour les phéochromocytomes bénins d'allure sporadique voire même d'une façon définitive dans les forme à haut risque de récidive et de malignité?

**Figure 1:**
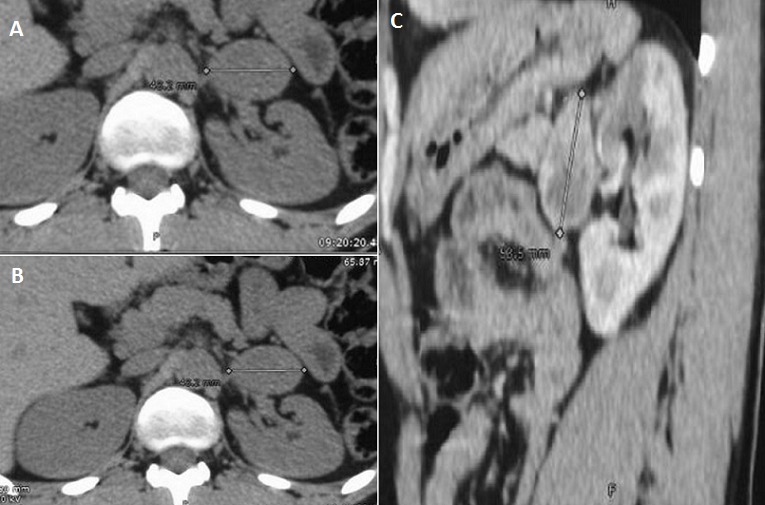
A) TDM abdominale en coupe axiale sans injection de produit de contrast; B)TDM abdominale en coupe axiale avec injection de produit de contrast; C)TDM abdominale en coupe sagittale avec injection de produit de contrast

